# Clinical image of omphalocele: a rare congenital defect of the abdominal wall

**DOI:** 10.11604/pamj.2024.47.115.42851

**Published:** 2024-03-11

**Authors:** Swati Rathod, Vaishali Taksande

**Affiliations:** 1 Department of Obstetrics and Gynaecological Department, Smt. Radhikabai Meghe Memorial College of Nursing, Datta Meghe Institute of Medical Sciences, Sawangi, Wardha, Maharashtra, India

**Keywords:** Omphalocele, abdominal wall defect, gastrochisis, exomphalos

## Image in medicine

We here report the case of a 1.8-kg baby girl with omphalocele born naturally via vaginal birth to a primipara mother at 37 weeks gestation. Omphalocele is a congenital malformation of the abdominal wall in which the organs of the abdomen stick out through an opening in muscles in the area of the umbilical cord. These organs are covered by a transparent membrane. The patient underwent a 2d ultrasound which revealed biventricular hypertrophy, intact ventricular septum, small patent foramen ovale with left-to-right shunt, and good biventricular systolic function. Trisomy 18 is the most frequent abnormality associated with omphalocele (22% to 89% of fetuses having omphalocele) followed by trisomy 13. Survival rates for babies who have an omphalocele and serious problems with other organs are about 70 percent. Omphalocele occurs when the gut contents fail to rotate and return to the abdominal cavity after normal embryonic herniation into the umbilical cord during weeks 6-10 of development. This study involved patients with a minor omphalocele (a defect measuring ≤4 cm) or a giant omphalocele. In the case of untreatable omphalocele, the intestine which remains outside the abdomen is 90%. The evidence demonstrates the association between congenital omphalocele and maternal tobacco and alcohol consumption. Women consuming multivitamin pills, most of which include folic acid, should be informed of the risk of having children with omphalocele, which occurs while the baby is developing in the womb.

**Figure 1:**
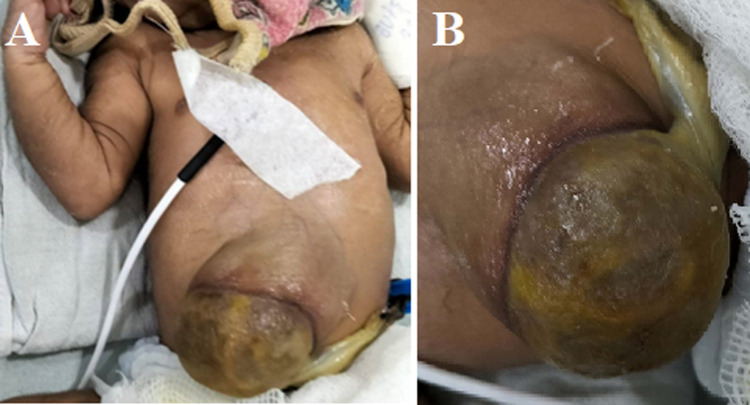
(A,B) intestinal and abdominal wall extended outside the abdomen

